# Prolonged viral shedding of SARS-CoV-2 in two immunocompromised patients, a case report

**DOI:** 10.1186/s12879-021-06429-5

**Published:** 2021-08-03

**Authors:** Melissa Niyonkuru, Rune Micha Pedersen, Kristian Assing, Thomas Emil Andersen, Marianne Nielsine Skov, Isik Somuncu Johansen, Lone Wulff Madsen

**Affiliations:** 1 Department of Infectious Diseases, Odense University Hospital, J.B Winsløws Vej 4, 5000 Odense C, Denmark; 2 Research Unit for Infectious Diseases, University of Southern Denmark, Odense, Denmark; 3 Department of Clinical Microbiology, Odense University Hospital, Odense, Denmark; 4 Research Unit for Clinical Microbiology, University of Southern Denmark, Odense, Denmark; 5 Department of Clinical Immunology, Odense University Hospital, Odense, Denmark

**Keywords:** SARS-CoV-2, Immunocompromised host, Virus shedding, COVID-19

## Abstract

**Background:**

The duration of viable Severe Acute Respiratory Syndrome Coronavirus 2 (SARS-CoV-2) shedding in immunocompromised patients is still unknown. This case report describes the duration of viable SARS-CoV-2 in two immunocompromised patients with completely different clinical courses and further addresses the immunological aspects.

**Case presentations:**

Oropharyngeal swaps were collected continuously during hospitalization for two immunocompromised patients infected with SARS-CoV-2 and sent for analysis to real time

reverse transcription polymerase chain reaction (RT-PCR), viral culture assessed by plaque assay and full genome sequencing. Blood samples for flow cytometry and further immunological analysis were taken once during admission. One patient was without symptoms of Coronavirus disease 2019 (COVID-19) whereas the other had severe respiratory symptoms requiring a stay at an intensive care unit (ICU) and treatment with remdesivir and dexamethasone. Despite their difference in clinical courses, they both continuously shed SARS-CoV-2 with high viral loads in culture. Both patients had undetectable anti SARS-CoV-2 IgG levels about 2 weeks after the first positive real time RT-PCR test of SARS-CoV-2, marked expansions of virus reactive CD8+ T cells but cellular markers indicative of attenuated humoral immunity.

**Conclusions:**

Our case illustrates the importance of distinguishing isolation guidelines for patients infected with SARS-CoV-2 according to their immunological status. Furthermore, it demonstrates the need for immune markers relating to viral shedding in immunocompromised patients.

**Supplementary Information:**

The online version contains supplementary material available at 10.1186/s12879-021-06429-5.

## Background

The need to determine the duration of Severe Acute Respiratory Syndrome Coronavirus 2 (SARS-CoV-2) shedding in infected individuals and thereby estimate the period of infectiousness is of vital importance in containing the coronavirus disease 2019 (COVID-19) pandemic.

Measured by real time reverse transcription polymerase chain reaction (RT-PCR), infected patients shed virus for up to 83 days after symptom onset, but replicative virus were not detected by culture after 9 days of symptoms [1]. A study including 21 hospitalized COVID-19 patients showed that the median time from symptom onset to viral clearance in culture was 7 days [2]. These findings underlie the isolation guidelines used by the World Health Organization (WHO) which state that SARS-CoV-2 infected persons can discontinue isolation 10 days after symptom onset plus at least 3 additional days without symptoms [3]. Regarding asymptomatic cases with SARS-CoV-2 infection, the duration of infectiousness is still uncertain. Asymptomatic patients appear to shed virus for longer periods than symptomatic patients, measured by real time RT-PCR, and this may reflect a weaker degree of immune activation [4]. Jiang et al. [5] reported shortened IgG sero-conversion times among asymptomatic compared to symptomatic patients, whereas Shirin et al. reported the opposite [6]. These deviating findings could be attributable to differences in study groups, methods of symptoms recollection, viral loads and sensitivity of immune assays or underlying immunity. Immune markers might be useful as determinants for prolonged SARS-CoV-2 shedding. A limited number of case reports indicate protracted viral shedding in immunocompromised patients observed in both real time RT-PCR and in viral culture [7–9] but these studies provided no detailed description of cellular immune status.

Here we report two cases of immunocompromised patients in which one patient had a severe course of COVID-19 whereas the other patient was asymptomatic.

## Case presentations

### Patient 1

A 66-year-old male, newly liver transplanted, was hospitalized on November 9, 2 days after symptom onset due to increased fatigue and tachypnea. An oropharyngeal swap on October 26 revealed that he was positive for SARS CoV-2 after close contact to known infected person on October 20. The patient had noted a diminished sense of taste and smell from November 1th, but was otherwise asymptomatic prior to admission. He received mycophenolatmofetil 500 mg twice daily, tacrolimus 4 mg × 2 and prednisolone 15 mg × 1. At admission, his peripheral oxygen saturation (SpO2), blood gas analysis and C-reactive protein (CRP) concentration were normal. During admission mycophenolatmofetil was paused and tacrolimus was reduced in dose. Prednisolone dosage was increased to 55 mg daily shortly after admission. On day 12 from symptom onset, he was transferred to the intensive care unit (ICU) for non-invasive ventilation. At the ICU he was treated with intravenous dexamethasone 6 mg daily and remdesivir 200 mg × 1 the first day and 100 mg × 1 the following 4 days. He gradually improved with a total ICU admission of 8 days and total hospitalization of 29 days.

#### Viral culture

**Fig. 1:**
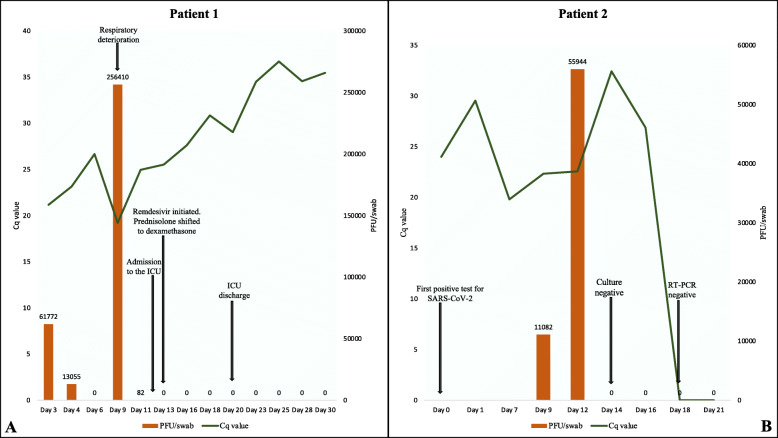
**A** Days after symptom onset with corresponding Cq and PFU/swab values for patient 1. Abbreviations: Cq value; PCR Quantification Cycle-value, PFU; Plaque Forming Unit, ICU; Intensive Care Unit. **B** Days from first positive test and corresponding Cq and PFU/swab values for patient 2. Abbreviations: PCR Quantification Cycle-value; Cq value, Plaque Forming Unit; PFU.

The first viral culture on SARS-CoV-2 was performed the day after admission correlating to 3 days after symptom onset and 21 days after exposure. This showed a PCR Quantification Cycle-value (Cq value) at 21.7 and viral culture at 61,277 Plaque Forming Unit (PFU)/swab. The latter rose to 256,410 PFU/swab on symptom day 9, with the Cq value diminishing to 19.21. From symptom day 13 virus were no longer detected in cultures and his Cq value gradually increased and was 35.45 before hospital discharge corresponding to day 31 from symptom onset (Fig. 1A). Full genome sequencing showed a B.1.177 linage [see Additional file 1].

### Patient 2

A 70-year-old male with multiple myeloma and previous autologous bone marrow transplantation. In July 2020, he had a pacemaker implanted. He received lenalidomide 25 mg daily. He was admitted on October 21 due to fever and elevated CRP concentration (130 mg/L). *Nocardia Farcinica* was detected in blood cultures and pus from abscesses on the left leg. Trans-esophageal echocardiography showed vegetation on the pacemaker electrode. The pacemaker was extracted and re-implanted after 6 weeks of antibiotic treatment for the disseminated *Nocardia* infection. Due to hospital policy, he was tested routinely every week for SARS-CoV-2 in real time RT-PCR during his 7-week long admission, and by week 4 the test was positive. He remained asymptomatic of the infection with SARS-CoV-2.

#### Viral culture

The first viral culture was done 9 days after the first positive real time RT-PCR test and plaque assay showed a viral load of 11,082 PFU/swab. Three days later, the viral load increased to 55,944 PFU/swab, despite no major clinical change, and with a steady Cq value of 22.33 and 22.57, respectively. Viral clearance in culture was observed after 12 days from the first positive real time RT-PCR test and after further 6 days, the real time RT-PCR test was negative (Fig. 1B). Whole genome sequencing showed a B.1.1.298 lineage described as a Danish lineage containing the origin of the Y453F mutation associated with mink [see Additional file 1].

#### Immunological results from patient 1 and 2

Both COVID 19 patients had normal concentrations of circulating immunoglobulins, neutrophils and monocytes [see Additional file 2, Table 1]. Both patients had undetectable anti-SARS-CoV-2 IgG levels < 1 RU/mL (QuantiVac IgG) 16 (patient1) and 14 days (patient2), respectively, after the first positive real time RT-PCR test of SARS-CoV-2.

Cryopreserved peripheral blood mononuclear cells (PBMC) were thawed and used for downstream applications: markedly reduced fractions (2% for both) of patients CD4+ T cells proliferated in response to allogeneic cells at day 6 although responding CD4+ T cells underwent a normal number of divisions [proliferation index, Additional file 2 Table 1] after allogeneic stimulation. Lymphocyte marker studies revealed expanded activated (HLA-DR+) CD3+ T cells and CD8+ CD38+ HLA-DR+ T cells in both patients. Frequencies of PD-1+ ICOS+ (% CD4+ CXCR5+) circulating T follicular helper cells (cT_FH_) among patients were comparable to those of controls and within normal range. Frequencies of CD19+ CD27+ CD38+ antibody secreting cells (ASC) were slightly elevated in patient 2 with myeloma [Additional file 2 Table 1].

## Methods

For detailed description of laboratory methods, see Additional file 3.

## Discussion and conclusions

Despite the clinical differences in these two immunocompromised patients, both continuously shed SARS-CoV-2 as measured by real time RT-PCR and had high viral loads in culture. To our knowledge, this is the first report describing the duration and amount of viable virus in an asymptomatic immunocompromised adult patient infected with SARS-CoV-2. Furthermore, not many studies have quantified viable virus. The significance of the amount of viable virus is still uncertain, but it must be assumed that higher viral load will mean greater infectivity.

Patient 1 shed viable virus until day 13 after symptom onset and 25 days after the first positive real time RT-PCR test. The real time RT-PCR was positive as far as 42 days after first positive test and remained positive during admission. This emphasizes that a positive real time RT-PCR not necessarily reflects replicative virus with implications for the estimation of SARS-CoV-2 transmissivity especially in immunocompromised patients. The asymptomatic patient had viable virus 12 days after initial positive real time RT-PCR. At that time point, he was highly contagious based on plaque assay analysis showing a high viral load of 55,944 PFU/swab. According to WHO, asymptomatic patients can discontinue isolation after 10 days of isolation from the first positive test [3]. This case shows that the immunocompromised patient, though asymptomatic, can potentially shed viable SARS-CoV-2 after 10 days and therefore this patient deviates from the guidelines.

Whole genome sequencing showed two different virus lineages and whether this can explain the different clinical courses of COVID-19 is unknown.

As IgG seroconversion times in asymptomatic/mildly symptomatic COVID-19 patients diverge [5, 6], cellular immune markers may represent underlying determinants for seroconversion and viral shedding. Both patients displayed markedly reduced fractions of allo-responsive CD4+ T cells. Allo-responsive T cells are dependent upon cognate TCR-MHC-peptide interactions as is the generation of cT_FH_ [10]. Patient 1 received a T cell repressive regimen while patient 2 was lymphopenic and received lenalidomid which suppresses CD4+ T cell proliferation in-vitro [11]. Patient 2 also suffered from a disseminated *Nocardia* infection, indicating compromised CD4+ T cell immunity. Hence, both patients reduced allo-reactive CD4+ T cell fractions were consistent with their impaired cT_FH_ formation versus that of an immunocompetent COVID-19 patient (day 20: 7.14%) [12]. CT_FH_ are strong indicators for the generation of antibodies to protein antigens [10]. Expansion of viral sensitive CD8+ CD38+ HLA-DR+ T cells [12] was observed in both patients, consistent with their preserved fractions of allo-reactive CD8+ T cells [Additional file 2 Table 1]. Frequencies of ASC were slightly elevated in patient 2 with myeloma but still lower than the ASC frequencies reported in symptomatic COVID-19, also during convalescence [12].

However, the surface markers CD27+, CD19+, and CD38+ are phenotypic characteristics associated with myeloma [13, 14], thereby questioning the immunological relevance of patient 2’s increased ASC frequencies. Compromised humoral immunity also characterizes myeloma patients. The strength of this case report is the inclusion of analyzes and description of cellular immune markers in both the asymptomatic and the symptomatic immunocompromised patient infected with SARS-CoV_2. Furthermore, the quantification of viable virus with plaque assay gives a more accurate picture of the duration of infectiveness. As a limitation, whole genome sequencing was only performed once during admission for both patients, and therefore potential mutations during hospitalization were not characterized.

In conclusion, both immunocompromised patients displayed marked expansions of virus reactive CD8+ T cells but cellular markers indicative of attenuated humoral immunity. Both patients shed high amounts of viable virus, despite completely different clinical courses and our cases indicate the importance of individual considerations relating to isolation for immunocompromised patients.

## Supplementary Information

**Additional file 1.** Sequence type for the two patients.

**Additional file 2: Table 1.** Immunological results for the 2 patients compared to controls.

**Additional file 3.** Methods.

## Data Availability

The dataset generated and analyzed for this cohort study is not publicly available due to the Danish Data Protection Law in accordance with approval by the Danish Data Protection Agency (j.nr. 20/16202).

## Abbreviations

SARS-CoV-2: Severe acute respiratory syndrome coronavirus 2
RT PCR: Reverse transcription polymerase chain reaction
COVID-19: Coronavirus disease 2019
ICU: Intensive care unit
Cq value: PCR quantification cycle-value
PFU: Plaque forming unit
PBMC: Peripheral blood mononuclear cells
cT_FH_: T follicular helper cells
ASC: Antibody secreting cells
