# Supplementary material for: Prolonged viral shedding of SARS-CoV-2 in two immunocompromised patients, a case report
Source: BMC Infect Dis. 2021 Aug 3;21:743. doi: 10.1186/s12879-021-06429-5 (PMC8330202; doi:10.1186/s12879-021-06429-5)
Supplement: Supplementary file 1 — Additional file 1. Sequence type for the two patients. [file 12879_2021_6429_MOESM1_ESM.docx]

**Additional file 1**

Sequence type for the two patients.

Patient 1: The sequence type for patient 1 showed a total of 7 amino acid substitutions (N:A220V,ORF1a:L450F,ORF1a:P3952S,ORF1b:P314L,ORF1b:L1681F,S:A222V,S:D614G) but no amino acid deletions.

Patient 2: The sequence type showed a total of 13 amino acid substitutions (N:N29H,N:S194L,N:R203K,N:G204R,N:A252S,ORF1a:N2082S,ORF1b:P314L,ORF1b:T730I,ORF1b:V2073L,ORF3a:H182Y,ORF9b:R25S,S:Y453F,S:D614G) and 4 amino acid deletions (ORF1a:M85-,ORF1a:S2083-,S:H69-,S:V70-).
