# Supplementary material for: Prolonged viral shedding of SARS-CoV-2 in two immunocompromised patients, a case report
Source: BMC Infect Dis. 2021 Aug 3;21:743. doi: 10.1186/s12879-021-06429-5 (PMC8330202; doi:10.1186/s12879-021-06429-5)
Supplement: Supplementary file 2 — Additional file 2: Table 1. Immunological results for the 2 patients compared to controls. [file 12879_2021_6429_MOESM2_ESM.docx]

**Additional file 2**

Table 1: Immunological results for the 2 patients compared to controls.

|  | P1 | P2 | C1 (m, 60+) | C2 (m, 60+) | In-house ref. |
| --- | --- | --- | --- | --- | --- |
| CRP (mg/L) | 46 | 7 | ND | ND | < 6 |
| IgA (g/L) | 1.1^#^ | 0.8 | ND | ND | 0.7-4.3 |
| IgG (g/L) | 11.9^#^ | 6.7 | ND | ND | 6.1-15.7 |
| IgM (g/L) | 0.87^#^ | 0.6 | ND | ND | 0.4-2.30 |
| Neutrophils (10^9^/L) | 2.4 | 3.6 | ND | ND | 1.5-7.5 |
| Lymphocytes (10^9^/L) | 1.2 | **0.6** | ND | ND | 1.0-4.0 |
| Monocytes (10^9^/L) | 0.22 | 0.6 | ND | ND | 0.20-0.80 |
| SARS-Cov-2 quant.(RU/mL) | < 1 | < 1 | ND | ND | < 1 |
| CD4+ allo (RF%) | **2** | **2** | 13^¤^ | 11 | 4-14 |
| CD8+ allo (RF%) | 5 | 6 | 14^¤^ | 5 | 5-20 |
| CD4+ allo (PI, divisions) | 3 | 4 | 4^¤^ | 4 | 3-5 |
| CD8+ allo (PI, divisions) | 4 | 4 | 4^¤^ | 4 | 3-5 |
| CD4+ PHA (RF%) | 77 | 73 | 93^¤^ | 66 | 58-93 |
| CD8+ PHA (RF%) | 100 | 77 | 81^¤^ | 84 | 56-91 |
| CD4+ PHA (PI, divisions) | 6 | 5 | 5^¤^ | 5 | 3-5 |
| CD8+ PHA (PI, divisions) | 6 | 5 | 5^¤^ | 4 | 3-5 |
| HLA-DR+ (% CD3+) | 29 | 42 | 20 | 18 | ≤ 20 |
| CD38+ HLA-DR+ (% CD8+) | 24 | 19 | 6 | 3 | 1.47 ± 0.50** |
| PD-1+ ICOS+ cT_FH_ (% CD4+ CXCR5+) | 1.29 | 1.75 | 1.34 | 1.11 | 1.83 ± 0.77** |
| CD27^high^ CD38^high^ ASC (% CD19+) | 0.08 | 2.94 | 1.26 | 0.91 | 0.61 ± 0.40** |

Abbreviations: m = male, 60+ = age > 60 years, C = control, ND= not determined, RF = responding fraction, PI= proliferation index, cT_FH_ = circulating T follicular helper cells, ASC = antibody secreting cells, ^#^ 5 months prior to immunological work-up. ^¤^Another control than C1.** Average ± s.d., Thevarajan I *et al*. *Nat Med* 2020.

Both controls had not reported any COVID 19 related symtoms since debut of the pandemic and Control 1 was SARS-CoV-2 antibody negative while Control 2 was PCR screen negative shortly prior to blood sampling.
