# Supplementary material for: Prolonged viral shedding of SARS-CoV-2 in two immunocompromised patients, a case report
Source: BMC Infect Dis. 2021 Aug 3;21:743. doi: 10.1186/s12879-021-06429-5 (PMC8330202; doi:10.1186/s12879-021-06429-5)
Supplement: Supplementary file 3 — Additional file 3. Methods. [file 12879_2021_6429_MOESM3_ESM.docx]

**Additional file 3**

**Methods**

*Flow-cytometry*

All flow-cytometric antibodies were primary conjugated: CD3-PerCP, CD4-APC, CD4-PerCP, CD8-PE, CD8-PerCP, CD19-APC, CD27-PE, CD38-FITC, HLA-DR-APC, PD-1-PE, ICOS-BV421 and CXCR5-FITC and from Becton Dickinson (New Jersey, USA) except CD8-PerCP (Invitrogen, Thermo Fischer Scientific, Waltham, USA). A BD FACSCanto II was used.

*T cell proliferation*

Peripheral blood mononuclear cells (PBMC) were stained with carboxyfluorescein diacetate succinimidyl ester (CFSE) and stimulated (100.000 cells/well (96 well plate), concentration 1 x 10^6^ cells/mL) for 6 days with either PHA (1.5ug/well), irradiated allogeneic PBMC (200.000 cells/well) or left unstimulated. The percentage of dividing CD3^+^ (PerCP) CD4^+^ (APC) and CD3^+^ (PerCP) CD8^+^ (PE) T cells, at day 6, were determined by decreases in CFSE fluorescence in cells from three pooled wells per stimulation modality. Dead cells were ascertained by propidium iodide (PI) staining.

*Antibody essay*

The Quantivac IgG assay (Euroimmune, Lübeck, Germany) determines IgG directed against the S1 antigen including the receptor-binding domain (RBD) within a broad linear range. According to the manufacturer, the assay displays excellent correlation with the WHO reference material (First WHO International Standard for anti-SARS-CoV-2 immunoglobulin (NIBSC code:20/136)) and good agreement with various neutralization assays”

*Patient samples*

Each oropharyngeal swab sample was collected by trained personnel at the Department of Infectious Diseases at the Odense University Hospital, Denmark. Each swab was placed in a tube containing 3.5 mL of complete virus culturing media [DMEM-Glutamax (Gibco), 2% Heat inactivated FBS (biowest) 1%Pen-Strep (Gibco) and Amphotericin B 2.5 mg/L (Sigma)]. Within a timeframe of 4 hours samples were subjected to plaque assay, flask culturing and real time RT-PCR. The remaining sample material was snap frozen in liquid nitrogen and stored at -80°C.

*SARS-CoV-2 PCR assay*

RNA used for real-time RT-PCR was extracted from 300 µl oropharyngeal swab sample using Chemagic^TM^ 360 with the extraction kit Chemagic viral DNA/RNA 300 kit H96. Elution volume was 50 µl. The laboratory developed real-time PCR E gene assay used for SARS-CoV-2 detection has been described previously [1]. This assay targeted a conserved sequence in the E gene region that is shared by the Sarbecovirus subgenus group. Real-time RT-PCR was performed in 20 µl reactions containing 5 µl 4 X TaqMan Fast Virus 1-Step master mix (ThermoFisher) with 1000 nM of each primer and 200 nM of the probe, and 6 µl RNA eluate. An internal RNA control (Newcastle disease virus vaccine strain; MSD) was added to the sample prior to RNA extraction. Primer and probe sequences targeting the internal control virus were kindly provided by Kurt Handberg, Department of Clinical Microbiology, Aarhus University Hospital (NDV-FWD-2: 5'-CACTGTCGGCATTATCGATGA-3’, NDV-REV: 5'-GAGCATCGCAGCGGAAA-3’, NDV-Probe: 5'-FAM-CCCAAGCGCGAGTTA-MGB-3’). Reverse transcription and amplification were performed using Lightcycler 480 (Roche) in 96 well format. The cycling conditions were as follows: Reverse transcription at 50 °C for 5 min, inactivation of RT/initial denaturation at 95° C for 20 sec, followed by 45 cycles of 95°C for 15 sec, 60 °C for 1 min for amplification. Samples with obtained Cq values below 40 with acceptable curves was reported as positive.

*Full genome sequencing*

Viral RNA was extracted using Chemagic^TM^ 360 as described above and used as a template to amplify and sequence the SARS-CoV-2 genome. Briefly, cDNA was synthesized from 11 μL of viral RNA using the SuperScript IV First-Strand Synthesis System (Invitrogen, USA) with random hexamers. PCR was performed using Q5 Hot Start High-Fidelity DNA Polymerase (NEB, USA) and a set of primers targeting regions of the SARS-CoV-2 genome designed by the ARTIC network (https://artic.network/ncov-2019, V3). The PCR program was: Heat activation at 98 °C for 30 sec, followed by 30 cycles of 98 °C for 15 s and 65 °C for 5 min. Briefly, amplified PCR products were purified by 0,8x AMPure XP bead (Beckman Coulter, California, CA). Equal molar of each amplified PCR products were then subjected to DNA repair, end preparation, and native barcode ligation (EXP-NBD104, EXP-NBD114 Oxford Nanopore Technologies). Barcoded samples were pooled and were ligated to sequencing adaptor. Sequencing was performed with Oxford Nanopore MinION device using R9.4.1 flow cell for 4 hours. After sequencing, Guppy v4.0.9 was used in converting the raw signal data into FASTQ format, demultiplexing, removal of nanopore and SISPA adaptor sequences.

*Viral Culture*

Plaque assay

Plaque assays were performed in a modified version of a previously published protocol [2]. In brief, 300 µL of the original sample and up to five ten-fold dilutions of it, was each added to 24-hour cultures of Vero E6 cells (ATCC® CRL-1586™) in 6-well microtiter plates (Thermo Scientific). Plates were left to incubate one hour at 37°C and 5% CO2 on a rocking platform. Overlay agarose gel was added and the plates were incubated at 37°C and 5% CO2 in a humidified atmosphere for three days. At termination, 800 µL of 10% neutral buffered formalin (Sigma) was added to each well and left at room temperature for 1 hour. Overlay was removed and the wells were stained with 10% crystal violet solution (Sigma) and PFU were counted after 2 minutes.

Flask culture

In order to confirm detection of viable virions by plaque assay and enhance sensitivity 700 µL of the original sample material were added to flasks (25 cm^2^, Nunc) containing a 24-hour culture of Vero E6 cells. The flasks were incubated one hour at 37°C and 5% CO2 on a rocking platform. Additional 5 ml of virus culturing media was added to each flask and a sample of 650 µL was aspirated and subjected to real time RT-PCR. The flasks were then incubated at 37°C and 5% CO2 in a humidified atmosphere. After three days of incubation the flasks were inspected by phase-contrast microscopy for cytopathic effects and another 650 µL was aspirated and subjected to real time RT-PCR.

**References Additional file 3**

1. Corman VM, Landt O, Kaiser M, et al. Detection of 2019 novel coronavirus (2019-nCoV) by real-time RT-PCR. Euro Surveill **2020**; 25(3).

2. Leibowitz J, Kaufman G, Liu P. Coronaviruses: propagation, quantification, storage, and construction of recombinant mouse hepatitis virus. Curr Protoc Microbiol **2011**; Chapter 15(1): Unit 15E.1.
